# The History, present state, and future prospects of the Asian College of Psychosomatic Medicine (ACPM)

**DOI:** 10.1186/1751-0759-6-1

**Published:** 2012-01-19

**Authors:** Hiroshi Ishizu

**Affiliations:** 1 Asian College of Psychosomatic Medicine, Professor Emeritus, (Mental Health, Psychosomatic Medicine), University of the Ryukyus Okinawa, Japan

**Keywords:** The Asian College of Psychosomatic Medicine (ACPM), The Asian Chapter of the International College of Psychosomatic Medicine (ICPM-AC), history, perspective

## Abstract

The Asian College of Psychosomatic Medicine (ACPM) was founded as the Asian Chapter of the International College of Psychosomatic Medicine (ICPM-AC) in Tokyo on April 12, 1982.

The first president was Hitoshi ISHIKAWA (Japan), the vice-presidents were Mahalingam MAHADEVAN (Malaysia) and Burton G.BURTON-BRADLEY (Papua- New Guinea), and the general secretary was Sueharu TSUTSUI (Japan).

Five years previously, preparation for creation of the ICPM-AC was started at the 4^th ^World Congress of the International College of Psychosomatic Medicine (ICPM) held in Kyoto, Japan, September 5-9, 1977.

The First Congress of the ICPM-AC was held by the President Yujiro IKEMI in Tokyo on May 19-20, 1984. The main members in the early stage were Y. IKEMI, H. ISHIKAWA, S. TSUTSUI, Taisaku KATSURA, Tetsuya NAKAGAWA. Hiroyuki SUEMATSU and others from Japan and Hsien RIN (Taiwan), Seock Young KANG (Korea), M. MAHADEVAN. B.G. BURTON-BRADLEY and others from other Asian countries.

Thereafter, academic congresses of the ICPM-AC, the 2^nd ^to the 9^th^, were held approximately every two years, in Japan, India, Malaysia, Taiwan, Korea, and China. The name was changed to the Asian College of Psychosomatic Medicine (ACPM), and the 10^th ^to 14^th ^congresses were held in Taiwan, Okinawa (Japan), Australia, Korea, and China.

The current president of the Executive Board of ACPM is Chiharu KUBO, the Director of Kyushu University Hospital.

The next academic congress is the 15^th ^ACPM and will be hosted by Tserenkhuugyin LKHAGVASUREN in Ulaanbaatar, Mongolia from August 24-26, 2012.

Participating countries have expanded to include Asian-Oceanic countries such as Mongolia, Micronesia, Australia and Sri Lanka.

The main themes of the congresses have focused on psychosomatic disorders, culture - bound syndromes, oriental medicine, etc... To date,"Health promotion"by raising the level of mental health based on psychoneuroendocrinoimmunomodulation has been very important. Prevention is also important in the Asia - Oceana area, from the viewpoints of both psychosomatics and culture.

Above all, an awareness of existential, authentic health is a sure way to promote healthy longevity and psychosomatic well - being.

To pursue happiness and well-being subjectively, objectively, and ecologically will be the most important purposes of ACPM in the future.

## Introduction

November, 2009 marked the 50^th ^anniversary of the Japanese Society of Psychosomatic Medicine, and a grand 50^th ^commemorative ceremony was held at the Tokyo International Forum on June 5^th^, 2009. The following year, on March 31^st^, 2010, the "Fifty Years of the Japanese Society of Psychosomatic Medicine Annals" was published [1]. Within the annals, articles on the ceremony and an enormous number of documents on our society produced over the 50 year period were published. At that time, Hiroyuki SUEMATSU, chairman of the special committee for the compilation of the annals, requested the author to write about the Asian College of Psychosomatic Medicine (ACPM) for the annals, as it was an important, international congress. Chiharu KUBO, the President of the ACPM and the Director of Kyushu University Hospital, provided information about the time and locations of the 1^st ^through 14^th ^Congresses and a list of the presidents and their profiles [1]. Professor Hiroyuki SUEMATSU again requested at the 51^st ^Congress of the Japanese Society of Psychosomatic Medicine, held in Sendai on June 25^th^, more detailed descriptions of the circumstances of the congresses of ACPM and their contribution to the Japanese Society of Psychosomatic Medicine journal "Psychosomatic Medicine", because they would be valuable resources for the future. With Professor KUBO's consent, I, the Vice-President of the ACPM, took charge of compiling the contributions.

Fortunately, I was able to observe the organization of the Asian Chapter of the ICPM at the 4^th ^World Congress of the International College of Psychosomatic Medicine (ICPM) held in Kyoto in 1977, and I was also a presenter at the 1^st ^Congress of the Asian Chapter of the International College of Psychosomatic Medicine (ICPM-AC) held in Tokyo,1984. There was an Asian Chapter Establishment Committee Meeting with plenary lectures held between these two conferences in Tokyo on April 12^th^, 1982; however, I did not have information of this event. Therefore, I started inquiring and trying to collect information regarding it as well as other areas that did not have sufficient information. The notes of the Establishment Committee Meeting with the plenary lectures could not be found at the ACPM executive office, located at Kyushu University Hospital, and it was difficult to contact the people who were involved in the event. Thus, I sent inquiries to Professor A. N. Singh, in Canada, but little information was obtained. While I was struggling to find this information, with the strong cooperation of Professor Koji TSUBOI at Toho University School of Medicine I was able to get in contact with Professor Emeritus Sueharu TSUTSUI, who was the first General Secretary of the ICPM-AC. Finally, the information necessary to write this article was obtained in this way. I would like to give special thanks to Hiroyuki SUEMATSU, the President of the "Fifty Years of the Japanese Society of Psychosomatic Medicine Annals"; Chiharu KUBO, the ACPM President; the ACPM executive office at Kyushu University Hospital; Emeritus Professor Sueharu TSUTSUI; Professor Koji TSUBOI, and the many others who assisted in the writing of this article.

### The Asian College of Psychosomatic Medicine (ACPM)

#### Former Name: The Asian Chapter of the International College of Psychosomatic Medicine (ICPM-AC)

The Asian College of Psychosomatic Medicine (ACPM) originally started as The Asian Chapter of the International College of Psychosomatic Medicine (ICPM-AC). The name ICPM-AC was used for the 1st to the 9th Congresses, and the present name, ACPM, has been used since the 10th Congress.

#### 1. Establishment

When the 4^th ^World Congress of the International College of Psychosomatic Medicine (ICPM)[1,2] was held in Kyoto on September 5^th ^to 9^th^, 1977, a movement to establish an Asian Chapter was begun and a preparatory committee was set up.

The ICPM-AC Establishment Committee Meeting was held in Tokyo on April 12^th^, 1982. At that time, memorial lectures were given by four distinguished psychosomatists; Yujiro IKEMI (Japan), Alfred M. FREEDMAN (USA), Jinichi SUZUKI (Japan) and Masamoto HIGUCHI (Japan). The President Hitoshi ISHIKAWA (Japan), the Vice Presidents Burton G. BURTON-BRADLEY (Papua-New Guinea) and Mahalingam MAHADEVAN (Malaysia), and the General Secretary Sueharu TSUTSUI (Japan) took office, and Yujiro IKEMI was appointed Honorary President. It was decided that the ICPM-AC Congress would be held every two years in Japan or another Asian country.

The 1^st ^Congress of the ICPM-AC was hosted by President Yujiro IKEMI in Tokyo, Japan on May 19^th ^and 20^th^, 1984. The opening ceremony took place at Nippon Seinenkan, located in the Meiji Shrine outer garden. During the ceremony, Taisaku KATSURA, who was the General Secretary of the 1^st ^Congress of the ICPM-AC, took the role of master of ceremonies. First, President IKEMI gave a welcome address, which was followed by the greetings of ICPM-AC President Hitoshi ISHIKAWA, the Vice Presidents M. MAHADEVAN and B.G. BURTON-BRADLEY, and the General Secretary Sueharu TSUTSUI. ICPM President Chase P. KIMBALL delivered a congratulatory address and Masayoshi NAMIKI, the President of the 25^th ^Congress of the Japanese Society of Psychosomatic Medicine, gave a congratulatory address.

At the 1^st ^Congress of the ICPM-AC, plenary lectures were given by Jiro KANEKO, the Honorary President, and Yujiro IKEMI, the President. Also, eight special topics were presented, including those by Professor A.N. SINGH (Canada), Professor Hsien RIN (Taiwan), and Professor B. H. FOX (USA). In addition, 82 presentations were made on general topics. The first conference was a prosperous beginning, with participants from Japan, Taiwan, Korea, China, Malaysia, India, and Papua-New Guinea [3].

#### 2. Progress (Table 1, 2)

The 2^nd ^Congress of the ICPM-AC, a continuation of the 1^st ^Congress of the ICPM-AC held by conference president Yujiro IKEMI in Tokyo on May 19^th ^and 20^th ^, 1984, was hosted by President A.N. SINGH in New Delhi, India from December 12^th ^to 15^th^, 1986. The main theme of the conference was "Asian Perspective in Psychosomatic Medicine" and participants came not only from Japan, India, and Malaysia but also from Indonesia, Thailand, and Australia. Hitoshi ISHIKAWA passed the presidency on to Sueharu TSUTSUI. After the conference, the participants enjoyed sightseeing at the Taj Mahal, Bodh Gaya, and other wonderful places [4].

**Table 1:** Congress Presidents of the ICPM-AC and ACPM

Conference Number	Year	President	Location	Dates
1^st^	1984	Yujiro IKEMI	Tokyo, JAPAN	May 19~20
2^nd^	1986	Amarendra Narayan SINGH	New Delhi, INDIA	November 12~15
3^rd^	1988	Mahalingam MAHADEVAN	Kuala Lumpur, MALAYSHIA	November 25~27
4^th^	1990	Jinichi SUZUKI	Sendai, JAPAN	April 20~21
5^th^	1992	Hsien RIN	Taipei, TAIWAN	September 4~6
6^th^	1994	Chiharu KUBO	Fukuoka, JAPAN	June 5~6
7^th^	1996	LIU Zengyuan	Dalian, CHINA	September 5~7
8^th^	1998	Byung I1 MIN	Seoul, KOREA	October 28~30
9^th^	2000	Sueharu TSUTSUI	Tokyo, JAPAN	September 29~30
10^th^	2002	Ming-Been LEE	Taipei, TAIWAN	September 28~29
11^th^	2004	Hiroshi ISHIZU	Okinawa, JAPAN	October 23~24
12^th^	2006	Lorraine DENNERSTEIN	Melbourne, AUSTRALIA	November 24~25
13^th^	2008	Bong Yul HUH Kyung Bong KOH	Seoul, KOREA	August31~ September1 September 10~12
14^th^	2010	ZHAO Zhifu	Beijing, CHINA	August 24~26
15^th^	2012	Tserenkhuugyin LKHAGVASUREN	Ulaanbaatar, MONGOLIA	

ICPM-AC: The Asian Chapter of the International College of Psychosomatic Medicine[1^st ^~ 9^th^]

ACPM: The Asian College of Psychosomatic Medicine[10^th^~]

**Table 2:** Official Board of The Asian College of Psychosomatic Medicine (2010-2012)

President	Chiharu KUBO (Fukuoka, Japan)
**Vice-President**	Lorrain DENNERSTIN (Melbourne, Australia) Bong Yul HUH (Seoul, Korea) Hiroshi ISHIZU (Okinawa, Japan) Ming-Been LEE (Taipei, Taiwan) LIU Zengyuan (Dalian, China) Mahalingam MAHADEVAN (Kulala-Lumpur, Malaysia) Byung I1 MIN (Seoul, Korea) Amarendra Narayan SINGH (Kingston, Canada) Sueharu TSUTSUI (Tokyo, Japan) Jun Shick WHANG (Seoul, Korea) ZHAO Zhifu (Beijing, China)

**Counselors**	Yukihiro AGO (Okayama, Japan) Kazuo HASEGAWA (Kawasaki, Japan) Shigeaki HINOHARA (Tokyo, Japan) M.MAJUMDER (Kaula-Lumpur, Malaysia) Tetsuya NAKAGAWA (Fukuoka, Japan) Hsien RIN (Taipei, Taiwan) Shridhar SHARMA (New Delhi, India) Itsuro SOBUE (Nagoya, Japan) Hiroyuki SUEMATSU (Tokyo, Japan)

**Directors**	Imaculada J. GONZAGA-OPTAIA (Pohnpei, Micronesia) Michio HONGO (Sendai, Japan) Toshio ISHIKAWA (Tokyo, Japan) Hiroshi ISMAEL (Kosrae, Micronesia) Tomifusa KUBOKI (Tokyo, Japan) Shih-Cheng LIAO (Taipei, Taiwan) Tserenkhuugyin LKHAGVASUREN (Ulaanbaatar, Mongolia) MA Hongkun (Harbin, China) Yoshihide NAKAI (Osaka, Japan)

**General Secretary**	Tetsuaki INAMITSU (Fukuoka, Japan)

**Auditors**	Katsutaro NAGATA (Tokyo, Japan) Shoji NAGATA (Kitakyushu, Japan) Hong Keun OH (Seoul, Korea)

The 3^rd ^Congress of the ICPM-AC was hosted by conference president M. MAHADEVAN in Kuala Lumpur, Malaysia from December 25^th ^to 27^th^, 1988. Its theme was the same as the 2^nd ^conference, "Psychosomatic Medicine Asian Perspective". Sultan Azlan SHAH (Perak State) was selected as the Honorary President of the 3^rd ^Congress [5].

The 4^th ^Congress of the ICPM-AC was hosted by conference president Jinichi SUZUKI in Sendai, Japan, on April 20^th ^and 21^st^, 1990. Its main theme was "Psychosomatic Medicine in Primary Care". At this conference, participants from Brazil joined for the first time [6].

The 5^th ^Congress of the ICPM-AC was hosted by conference president Hsien RIN in Taipei, Taiwan, from September 4^th ^to 6^th^, 1992. Its main theme was "Panic and Anxiety". President RIN, the head of the Department of Psychiatry, National Taiwan University College of Medicine, studied at the Hokkaido Imperial University School of Medicine in Japan. Because of this, he developed a strong relationship with Japan and held a special welcome reception program for Japanese guests with visit to memorial places at the medical school of the former Taihoku Imperial University [7].

The 6^th ^Congress of the ICPM-AC was hosted by conference president Chiharu KUBO in Fukuoka, Japan on June 5^th ^and 6^th^, 1994. Its main theme was "Psychosomatic Medicine Symposium-East and West". Many participants and speakers came from China and Professor Wolfram SCHÜFFEL from Germany joined as a special participant [8].

The 7^th ^Congress of the ICPM-AC was hosted by conference president LIU Zengyuan in Dalian, China from September 5^th ^to 7^th^, 1996 with many Chinese participants. After the conference, the participants visited Hill 203 in Ryojun (Lushun) where one of the fiercest battles of the Russo-Japanese War was fought [9].

The 8^th ^Congress of the ICPM-AC was hosted by conference president Byung Il MIN in Seoul, South Korea from October 28^th ^to 30^th^, 1998. The participants interacted with the staff of Kyunghee University, learned Korean history and culture, and visited a folk village and other special places [10].

The 9^th ^Congress of the ICPM-AC was hosted by conference president Sueharu TSUTSUI in Tokyo, Japan on September 29^th ^and 30^th^, 2000. Its main theme was "New Encounter between Mind and Body-Stress Management in the 21^st ^Century" and a large number of speakers gave presentations. At the Executive Board Meeting of this conference, the name "The Asian Chapter of the International College of Psychosomatic Medicine" (ICPM-AC) was changed to "The Asian College of Psychosomatic Medicine"(ACPM) and this new name has been used since the 10^th ^Congress. Also, the presidency was handed over from Sueharu TSUTSUI to Chiharu KUBO [11].

The 10^th ^Congress of the ACPM was hosted by conference president Ming-Been LEE in Taipei, Taiwan on September 28^th ^and 29^th^, 2002. Starting with this conference, the official name of the conference became "The Asian College of Psychosomatic Medicine" (ACPM). Ten years had passed since the 5^th ^Congress was last held in Taiwan hosted by conference president Hsien RIN. The 2^nd ^conference in Taiwan was held at the same site as the 1^st ^conference. Its main theme was "Psychosomatic Medicine and Health Promotion" with about 250 participants. There were eight sessions of oral presentations and six poster sessions [12].

The 11^th ^Congress of the ACPM was hosted by conference president Hiroshi ISHIZU in Okinawa, Japan on October 23^rd ^and 24^th^, 2004. Its main theme was "Culture and Psychosomatic Medicine". The president's lecture, titled "Psychosomatic Medicine of Okinawan Culture and Healthy Longevity", as well as 6 special lectures, 1 symposium, 1 panel discussion, 2 educational lectures, 30 oral presentations, and 112 poster presentations were well received. The documents state that the symposium theme was chosen to be "Present and Future of ACPM" and all of the past presidents were invited. Also, for the future development of the ACPM, the congress president invited as special guest lecturers Professor Tserenkhuugyin LKHAGVASUREN, President of the National Medical University of Mongolia (now the National Health Sciences University of Mongolia) and Drs. Hiroshi ISMAEL and Imaculada J. GONZAGA-OPTAIA from the Federated States of Micronesia of the Pacific, who participated for the first time. Unfortunately, Professor Futa HELU of the Kingdom of Tonga could not participate due to other business matters. In addition, a professor in Kazakhstan was unable to be contacted. Despite these absences, the participants expanded our organization to include areas of the Asian-Pacific region from Mongolia in the north to Australia in the south and from Canada in the east to Sri Lanka in the west. In the Executive Board Meeting, the sites of the next four conferences were decided; The 12^th ^Congress at Melbourne, Australia, the 13^th ^Congress at Seoul, South Korea, the 14^th ^Congress at Beijing, China, and the 15^th ^Congress at Ulaanbaatar, Mongolia [13].

The 12^th ^Congress of the ACPM was hosted by conference president Lorraine DENNERSTEIN in Melbourne, Australia on December 24^th ^and 25^th^, 2006. The theme picked for the conference was noteworthy, "Sexual Health and Psychosomatic Medicine". The conference was successful due to of the tremendous support from Vice-President of the 12^th ^Congress, Koji TSUBOI, Professor of Psychosomatic Medicine at Toho University School of Medicine, Japan [14].

The 13^th ^Congress of the ACPM was hosted by conference president Bong Yul HUH in Seoul, South Korea on August 31^st ^and September 1^st^, 2008. Ten years had passed years since the 8^th ^Congress held in South Korea hosted by President MIN. Due to the influx of South Korean pop culture into other areas, a large number of people came to the conference and many distinctive Korean features were included in the presentations at the well-received conference. Kyung Bong KOH played the role of co-President [15].

The 14^th ^Congress of the ACPM was hosted by conference president ZHAO Zhifu in Beijing, China from September 10^th ^to 12^th^, 2010. The China National Convention Center, a fantastic building that is located in the center of Beijing and that was renovated for the Beijing Olympics, was the site of the conference. More than 400 topics were presented. The conference was especially well organized. The participants had the opportunity to see traditional Chinese Opera at the welcome party, visit the Great Wall, a World Heritage site, where one could enjoy the distant view of Inner Mongolia, and visit the Ming Dynasty Tombs. President ZHAO's son, Dr. ZHAO Peng, who is studying abroad at the Department of Psychosomatic Medicine of Kyushu University, ably assisted President KUBO in preparing for the conference [16].

The 15^th ^Congress of the ACPM will be hosted for the first time in Mongolia at Ulaanbaatar by conference president Tserenkhuugyin LKHAGVASUREN from August 24^th ^to 26^th^, 2012. Members of ACPM in Japan are inviting people from far and wide to come and back up the conference as much as possible. The first Mongolian Workshop of Psychosomatic Medicine was held on October 20^th^, 2006, and the Mongolian Society of Psychosomatic Medicine was established recently [17]. We expect a large number of presenters from Japan to be at the conference next year to support this developing society.

Most recently, Indonesia announced its candidacy for the 16^th ^Congress of the ACPM. The Indonesian Society of Psychosomatic Medicine, also newly established in 2010 in Jakarta, has its center at the National Indonesian University. For these newly established Asian societies of Psychosomatic Medicine to hold the conferences, it is important that as many of our members participate and present as many topics as possible. We would especially encourage the members of the Japanese Society of Psychosomatic Medicine to do all they can to assist these new societies.

## Conclusion

It is time to conclude this overview of the history and perspectives of the ACPM. When the ACPM began as the ICPM-AC, the members of ICPM from the United States, Canada, and other countries joined the conferences to support the developing East Asian Societies of Psychosomatic Medicine, such as those in South Korea, Taiwan, China, and Japan, which have become the core Asian societies. However, gradually all of the countries involved in the East Asian Societies of Psychosomatic Medicine had become highly developed. In the near future, our goal is to support the newly established and developing Societies of Psychosomatic Medicine of Mongolia, Indonesia, and other countries and we must do our best to foster the Societies of Psychosomatic Medicine of the Pacific Islands and other smaller countries. Psychosomatic Medicine is "The Medical Science of Happiness" which brings about human health in a true sense. We would like to ask all of the members of the Japanese Society of Psychosomatic Medicine for their support in assisting the developing Societies of Psychosomatic Medicine.

The ACPM has no official published journal, but publishes a booklet of the abstracts of presentations given at each Congress. Each Congress is well structured and abstracts are published at the beginning of every conference. Unfortunately, the abstracts are not available to everyone; thus, we have contrubuted this article containing data about the ACPM to *Bio-Psycho-Social Medicine *so that other members of the Societies of Psychosomatic Medicine outside of East Asia can better know what has been happening here and in the Oceanic Area.
